# Acute compartment syndrome resultant from alcohol abuse

**DOI:** 10.1093/jscr/rjab128

**Published:** 2021-04-22

**Authors:** Logan A Reed, Derek Miller, Angela Harbour

**Affiliations:** Florida State University College of Medicine, Tallahassee, FL 32306 USA

## Abstract

Acute compartment syndrome (ACS) is defined as a severe rise in pressure within a fascial compartment thereby leading to impaired microvascular perfusion of the limb. Therefore, ACS is a surgical emergency and if not treated immediately, permanent neurovascular and muscular compromise can ensue. When compartment syndrome is suspected, clinical recognition and timely assessment of the limb is essential to preventing limb ischemia. The classic cause of ACS is trauma; however, coagulopathies have been known to incite these events. Hemorrhage into a compartment can cause ACS but is rare in the literature. Here we present a case of thrombophilia resulting from pathological liver disease, leading to ACS that ultimately led to limb exsanguination. Education on early recognition of liver disease as an indirect underlying cause of ACS is imperative in order to prevent the dangerous sequelae that follow ACS.

## INTRODUCTION

Acute compartment syndrome (ACS) is an increase in compartment extremity pressure that subsequently prevents microvascular perfusion. ACS is usually the result of a traumatic incident such as a crush injury, long bone fracture or arterial thrombosis; however, in cases without a traumatic origin, a diagnostic dilemma is common [1]. The most common cause of ACS is fractures, accounting for ~69–75% of cases with the remaining few accounted for by ischemic events, iatrogenic procedures, idiopathic causes and rarely coagulopathies [2, 3]. Compartment syndrome can be seen in patients with hemophilia or while on anticoagulation therapy; however, atraumatic hypocoagulable states causing ACS due to alcoholic hepatitis are rare in the literature and more importantly, difficult to diagnosis in a timely manner [4, 5]. Emergent fasciotomy is required to relieve the pressure within the compartment and prevent tissue necrosis regardless of the risk of bleeding. Acute emergency visits often focus on diagnosis and treatment of ACS rather than the discovering the underlying cause of the pathology. Awareness is needed for providers with any patient presenting with a history of chronic alcohol use and signs of ACS in order to bridge the gap in lack of knowledge between this rare phenomena especially given the lack of a generalized comprehensive workup for a history of significant alcohol use in an acute care setting. Here we present an atypical cause of ACS in a patient with alcoholic liver cirrhosis that subsequently experienced a lower extremity hemorrhage secondary to a hypocoagulable state from undiagnosed liver disease.

**Figure 1:**
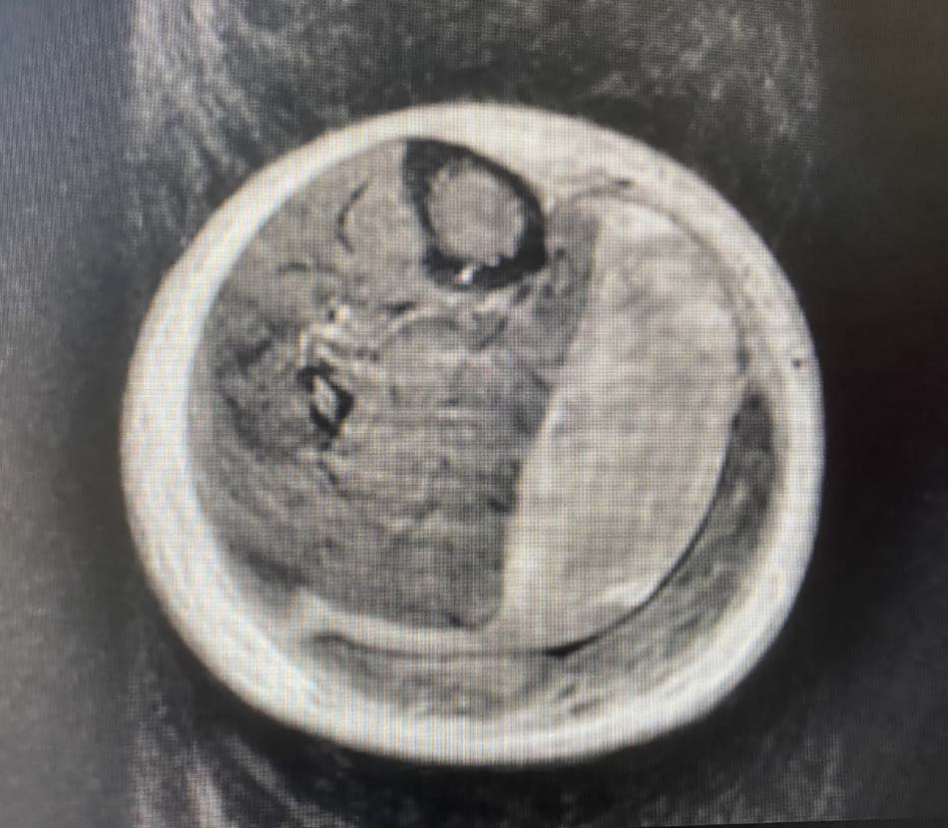
MRI of the RLE showing hematoma in the superficial posterior compartment.

## CASE REPORT

A 57-year-old female patient with history of alcohol abuse of 20 years presented to the emergency department on her own with right lower extremity (RLE) edema and pain of 1 week. She described a 5/10 pain exacerbated by ambulation that gradually worsened throughout the week. A detailed history failed to reveal any source of trauma. Patient denies any forms of intense physical exercise. She denies any other complaints. Patient was very pleasant and answered questions appropriately. No signs of acute encephalopathy were present. Palpation of the RLE revealed diffuse tenderness. Pulses were two plus bilaterally and active range of motion of the toes, ankle and knee was intact bilaterally. Passive range of motion produced 8/10 pain in RLE. Patient was unable to ambulate secondary to pain. Left extremity had normal active and passive range of motion without pain. No signs of effusion or erythema to the ankle, knee or foot bilaterally. Laboratory diagnostic blood studies suggested anemia (Hemoglobin 7 g) and liver disease (aspartate aminotransferase 420, alanine aminotransferase 200 and International normalized ratio of 2.9). Urgent RLE bedside ultrasound revealed complex fluid collection within the RLE. X-ray was unremarkable. Magnetic resonance imaging (MRI) (Fig. 1) was obtained and displayed a large hematoma at the intermuscular fascial plane between the medial head of the gastrocnemius and soleus related to a partial tear of the medial head of the gastrocnemius. Pressure recordings from handheld manometer confirmed the diagnosis of ACS that exhibited pressures of 72 and 74 mm Hg in the medial and superficial posterior compartments, respectively. Patient was then prepped and transported to the Operating room for emergent fasciotomy. A 25-cm incision was made on the lateral side of the RLE that did not reveal indication of true tissue necrosis. Dissection was continued deep to the tibia, allowing exposure of the fascial compartments of the intermuscular septum between the tibia and fibula. No indication of tissue necrosis was suspected and there was no sign of a hematoma in this area. A 25-cm medial incision was then performed with immediate hemorrhaging occurring upon release. In total, 75 ml of obvious hematoma was evacuated and macerated muscular tissue was identified. Closure was planned for a later date given the swelling associated and wound vacuum was placed. The patient was then transferred to the intensive care unit where stay was complicated by hypotension requiring pressor support and the development of a renal and hepatic failure due to shock. She was placed on temporary dialysis. Unfortunately, it was determined that the patient could not tolerate dialysis and she was later admitted to hospice care.

## DISCUSSION

Hemorrhage into the compartment of an extremity resulting from a hypocoagulable state is an unusual cause of ACS especially when attributed to liver failure. Although a medial gastrocnemius tear was diagnosed on MRI, the patient denied any form of an inciting event that raises the question to whether the patient may have been impaired and experienced an injury without recollection. Nonetheless, it is not surprising that atraumatic presentations of compartment syndrome present with a diagnostic dilemma and are often misdiagnosed or undiagnosed [6]. These atraumatic presentations may lead to a delay of diagnoses of up to 13 hours [7]. Consideration for ACS should always be on the differential when individuals present with diffuse extreme extremity pain in addition to hypocoagulable states such as liver disease, inherited coagulopathies or anticoagulation therapy, regardless of traumatic history. Any risk factors that lead to thrombophilia are potential sources for ACS and should be identified in suspected ACS.

In conclusion, a broad knowledge of the atypical presentations of ACS such as coagulopathies may aid in the early identification of ACS, thereby preventing limb necrosis.
